# Behaviour of Impurities during Electron Beam Melting of Copper Technogenic Material

**DOI:** 10.3390/ma15030936

**Published:** 2022-01-26

**Authors:** Katia Vutova, Vladislava Stefanova, Vania Vassileva, Milen Kadiyski

**Affiliations:** 1 Institute of Electronics, Bulgarian Academy of Sciences, 1784 Sofia, Bulgaria; vvvania@abv.bg; 2 Department of Metallurgy of Non-Ferrous Metals and Semiconductors Technologies, University of Chemical Technology and Metallurgy, 1756 Sofia, Bulgaria; vps@uctm.edu; 3 Aurubis Bulgaria AD, Industrial Zone, 2070 Pirdop, Bulgaria; m.kadiyski@aurubis.com

**Keywords:** copper technogenic material, electron beam, thermodynamic analysis, removal efficiency

## Abstract

The current study presents the electron beam melting (EBM) efficiency of copper technogenic material with high impurity content (Se, Te, Pb, Bi, Sn, As, Sb, Zn, Ni, Ag, etc.) by means of thermodynamic analysis and experimental tests. On the basis of the calculated values of Gibbs free energy and the physical state of the impurity (liquid and gaseous), a thermodynamic assessment of the possible chemical interactions occurring in the Cu-Cu_2_O-Me_x_ system in vacuum in the temperature range 1460–1800 K was made. The impact of the kinetic parameters (temperature and refining time) on the behaviour and the degree of removal of impurities was evaluated. Chemical and metallographic analysis of the obtained ingots is also discussed.

## 1. Introduction

Copper continues to be one of the most important metals that are at the basis of the economic development of society and the human efforts to achieve a higher standard of living [1]. This is due to its unique physical, mechanical and chemical properties. At present, approximately 50% of the copper in Europe is produced by recycling. Copper recycling is becoming one of the main methods of producing copper, as it requires up to 85% less energy compared to the conventional production schemes [2].

The impurities content in secondary copper raw materials varies from 0.8% to 1.5% [3]. They usually contain a considerable amount of dissolved oxygen and sulphur and metal impurities such as lead, tin, iron, nickel, arsenic, antimony, zinc, bismuth, selenium, tellurium, gold, silver and others [3].

According to [4], copper-soluble impurities (such as Al, Sn, Zn, etc.) increase the mechanical properties but significantly reduce the electrical and thermal conductivity. Insoluble impurities (such as Pb, Bi) form eutectics which melt at lower temperatures, which worsen the hot treatment of copper under pressure. Non-metallic impurities (such as S, O) form eutectics which melt at higher temperatures that are separated at the boundaries of copper grains. This in turn leads to the appearance of brittleness of copper. Impurities that form intermetallic compounds (such as Se, Te) are separated as intermediate phases at the grain boundaries, leading to brittleness.

In traditional metallurgy, the process of refining Cu scrap is carried out by either pyrometallurgical or hydrometallurgical method [5]. In the pyrometallurgical scheme, the removal of impurities is done in anode furnaces and by electrolysis. According to the authors, about 85% of anode copper is subjected to electrolytic refining [5]. In hydrometallurgical schemes, the copper scrap is first dissolved in acids and then recovered, for example, by liquid phase extraction [6,7].

The requirements for the quality of copper, its chemical composition and structure are constantly increasing with the development of new branches of energy and electronics [8]. This necessitates the search for new, effective methods of melting.

Methods such as laser, plasma and electron beam melting (EBM) are successfully applied following the development of modern metallurgy and effective methods for refining metals and alloys [9,10,11]. Of these methods, the EBM method is particularly appropriate as it combines the advantages of vacuum and high-energy special electrometallurgy [12,13,14,15,16]. Under vacuum conditions, some reactions take place that are impossible at atmospheric pressure. The electron beam (heat source) and the high vacuum ensure degassing and a high degree of purification of the material, as well as uniformity of the chemical composition and homogeneous structure of the obtained ingots. Lack of additional impurities originating from the used water-cooled copper crucible and the ability to control the energy of the heat source regardless of the material and the size of the feedstock are additional important benefits of the method.

There are a number of publications in the literature related to the thermodynamics and kinetics of copper refining from impurities at atmospheric pressure [5,17,18,19]. Results of the modified pyrometallurgical processing of waste printed circuit boards were presented in [18], while pyrometallurgical refining of copper in an anode furnace was studied in [5] in order to improve the process. The behavior of tin and antimony was experimentally studied in secondary copper pyrometallurgical smelting conditions [19]. According to [17], depending on the degree of removal of impurities in the anodic refining, they can be divided into three groups: Group I–impurities separated relatively easily and completely (such as Fe, Co, Pb, Sn, S); Group II–impurities partially separated (such as As, Sb, Ni); Group III–impurities (such as Ag, Au, Se, Te, Bi) separated to a negligible extent.

This work is a continuation of the research conducted so far by the team [20]. In [20], the investigated material contained 99.83% copper and the influence of the beam power and duration of melting process on the purity of the refined material was studied, the electron affinity to oxygen of the investigated impurities and the vapor pressures of metallic impurities and their oxides were evaluated as well. The aim of this work is to study the behaviour of impurities (metallic and non-metallic) and the influence of thermodynamic and kinetic technological parameters (temperature of thermal treatment, refining time) on the refining efficiency and the structure of the obtained copper during EBM of copper technogenic material (99.45%). On the basis of the calculated values of Gibbs free energy under EBM conditions, a thermodynamic assessment of the possible chemical interactions occurring during the melting and refining of copper technogenic material with high impurity content (Se, Te, Pb, Bi, Sn, As, Sb, Zn, Ni, Ag, O, etc.) was made and the physical state of the base metal and metallic impurities was taken into account.

## 2. Materials and Methods

**Figure 1:**
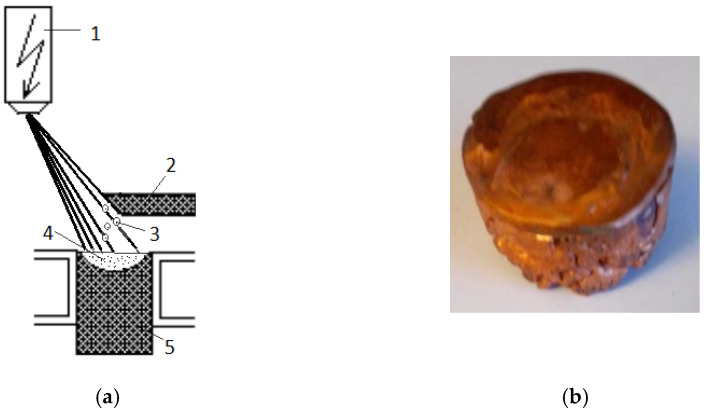
(**a**) Principal scheme of the EBMR process: (1) electron optical system; (2) started metal rod; (3) generated droplets; (4) molten pool in the water-cooled crucible; (5) metal ingot; (**b**) fabricated copper sample.

The experiments for copper technogenic material melting were performed using EBM installation with power 60 kW (ELIT-60) at the Physical problems of the EB technologies laboratory of the Institute of electronics, Bulgarian Academy of Sciences. ELIT-60 (Leybold GmbH, Cologne, Germany) is equipped with one electron gun (accelerating voltage of 24 kV), a feeding mechanism for horizontal input of the raw material, an extraction system (pulling mechanism), a water-cooled copper cylindrical crucible (a diameter of 50 mm) with moving bottom, where the molten metal solidifies and a circulation water cooling system–Figure 1. The operation vacuum pressure in the melting chamber is 3–6 × 10^−3^ Pa.

The copper content in the investigated technogenic material is 99.45% Cu (anode residues after an electrolysis process). The impurities with higher concentrations are: O (0.2251%), Ni (0.1%), As (0.07%), Se (0.0382%), Pb (0.021%), Bi (0.015%) and Ag (0.014%). With lower content are: S (0.0028%); Sb (0.0095%), Te (0.0068%), Sn (0.0034%) and Zn (0.0023%). The lowest is the content of Co, Cd, Fe, Au, etc. (<20 ppm), therefore these impurities are not taken into consideration in the further analysis.

The raw (initial) material was melted under single processing at melting powers of 6 kW (T = 1460 K), 7 kW (T = 1500 K), 13 kW (T = 1700 K) and 19.5 kW (T = 1800 K). At T = 1500 K the lengths of melting time are 15 min and 35 min, while at T = 1700 K the retention time is 20 min and 45 min. The melting time for T = 1460 K is 20 min and for T = 1800 K is 25 min, respectively. The raw materials mass was about 500 g (each sample). The chemical composition of the copper samples before and after EBM is determined with ARL 4460 OES Thermo Scientific spectrometer (Thermo Fisher Scientific, Waltham, MA, USA). The spectrometer is equipped with a Paschen-Runge vacuum polychromator working in argon atmosphere. Oxygen analyses of the samples were performed with ELTRA OH-900 oxygen/hydrogen determinator (Eltra GMBH, Haan, Germany).

A 4% solution of nitric acid in ethyl alcohol was used to etch and reveal the microstructure of the obtained metal specimens. The etching time was 30 s.

A light microscope Leica DM2500 (Leica Microsystems GmbH, Wetzlar, Germany) with a digital camera Leica EC3 (Leica Microsystems GmbH, Germany) was used for the metallographic study of the macro or micro-structure of polished and etched surfaces of copper samples. The image processing was performed using the Leica LAS software (Leica Microsystems GmbH, Germany).

## 3. Results

### 3.1. Thermodynamic Analysis of Possible Chemical Interactions during Electron Beam Melting and Refining (EBMR)

The thermodynamic analysis of the possible chemical interactions occurring during the refining of copper from impurities such as Se, Te, Bi, As, Sb, Pb, Sn, Ni, Zn, Ag under EBM conditions is performed on the basis of the Gibbs free energy (ΔF) and the physical state of the impurities. The analysis was carried out using the professional thermochemical calculation programme HSC Chemistry ver.7.1, module “Reaction Equation” [21], taking into account the physical state of copper and the metal impurities during EBM.

Since there is a constant pressure in the vacuum chamber during EBM, the main parameters that affect the refining process are the temperature of the metal and its physical state [9]. Another parameter that impacts the removal of impurities from the main metal is the mass transport of molten or solid metal particles to the reaction surface [22].

**Figure 2:**
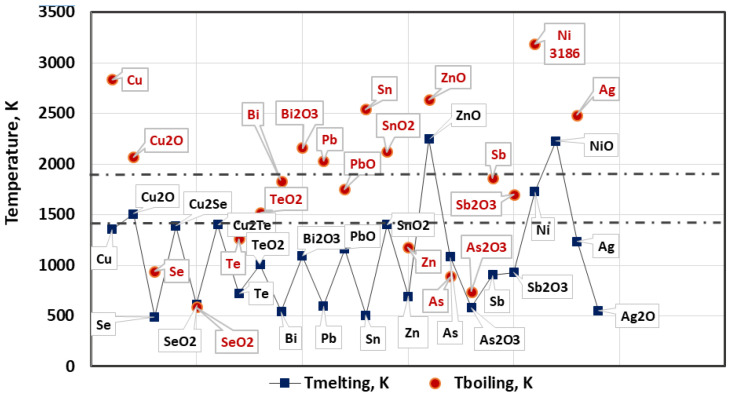
Melting and boiling temperatures of studied metals and compounds.

Figure 2 shows the melting and boiling temperatures of studied metals and compounds. The temperature range 1460–1800 K of e-beam melting process is marked in Figure 2 by dashed lines.

It can be seen that under vacuum conditions and the studied temperature range, the metal impurities present in Cu such as Pb, Bi, Sn, Sb, Ag and their oxides will be in a liquid state. Impurities such as Zn, As and its oxide have a boiling point significantly lower than the melting point of copper and they will be in the gaseous state. Figure 2 shows that the boiling points of Cu_2_O, Pb and Bi_2_O_3_ slightly exceed the operating temperature of 1800 K, which allows us to assume that they will also be in gaseous state under vacuum conditions.

Phase diagrams Cu-Se and Cu-Te show that both impurities are present in copper in the form of intermetallic compounds: copper selenides and tellurides [23,24]. At low selenium and tellurium content they are in the form of Cu_2_Se and Cu_2_Te. These compounds will be present in liquid state as they have a low melting point.

Ni and Ag remain in liquid state. Nickel has complete mutual solubility in copper and at 1358 K (the melting temperature of copper) it completely passes into liquid phase [25,26]. The melting temperature of ZnO, unlike Ni, is very high and under e-beam melting conditions ZnO will be present in solid state.

Therefore, under EBM conditions, the liquid metal is a complex system of Cu, Cu_2_O, metal impurities and their oxides, and they are in liquid, solid or gaseous state depending on the thermodynamic conditions of refining and the type of impurity.

Under e-beam melting conditions, the refining processes take place mainly on the reaction surfaces of the liquid metal (its interface with the vacuum, Figure 1) in three reaction zones [9]. Depending on the thermodynamic conditions of the EBM and the type of the individual impurities, the refining process can take place by: (i) degassing (removal of components, with a higher partial pressure than the partial pressure of the base metal), (ii) distillation (evaporation of the more volatile compounds from the metal components). Effective refining requires the implementation of the following inequalities concerning vapor pressures (p) of copper and the metallic impurities (R_i_): (p_RiO_) > (p_Ri_) > (p_Cu2O_) > (p_Cu_).

Thermodynamic evaluation of the possible chemical interactions in the system Cu(l)-Cu_2_O(l)-R_i_(l,g) is made on the basis of the following equations:

2Cu(l) + [O] = Cu_2_O(l) + ΔF_T,Cu/Cu2O_, (1)

R_i_(l,g) + [O] = R_i_O(l,g) + ΔF_T,Ri/RiO_, (2)

Cu_2_O(s,l) + R_i_(l,g) = R_i_O(l,g) + 2Cu(l) + ΔF_T_, (3)

where ΔF_T,Cu/Cu2O_, ΔF_T,Ri/RiO_ and ΔF_T_ are the Gibbs free energies of the respective processes. The indices (s), (l) and (g) mean that the substance is in a solid, liquid or gaseous state, respectively. The calculations were performed under melting conditions: at temperatures of 1460 K, 1500 K, 1600 K, 1700 K and 1800 K and operating pressure in the vacuum chamber of 10^−3^ Pa. These parameters correspond to the actual conditions of melting and refining of copper technogenic material in the EBM plant.

**Figure 3:**
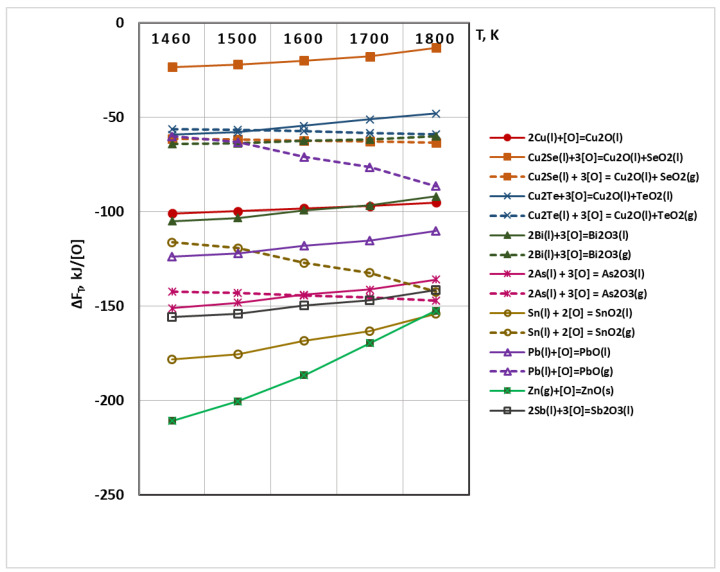
Influence of the temperature on ΔF_T_ of oxidation reactions of Cu(l) and R_i_(l,g) under vacuum conditions.

Figure 3 shows the temperature dependences of the free energies of oxidation of copper (ΔF_T,Cu/Cu2O_) and metal-impurities (ΔF_T,Ri/RiO_). The analysis of the obtained dependences shows that the probability of oxidation of ZnO(g) to ZnO(s) is the highest and as the temperature increases, the affinity of Zn(g) to oxygen decreases significantly. In the whole temperature range, the values of ΔF of formation of SnO_2_(l), Sb_2_O_3_(l), As_2_O_3_(l) and PbO(l) are higher than that of the oxidation of Cu(l) to Cu_2_O(l). The thermodynamic probability of oxidation of bismuth to Bi_2_O_3_(l) is almost the same as that of copper to Cu_2_O, while the probability of oxidation of copper telluride to TeO_2_(l) and copper selenide to SeO_2_(l) is significantly lower than that of copper.

It is observed that as the temperature increases, the values of the Gibbs energy of the oxidation of impurities: Sn(l) → SnO_2_(g) and Pb(l) → PbO(g) increase significantly but they are significantly lower than those of oxidation to liquid oxides.

Energy of oxidation of Bi(l) → Bi_2_O_3_(g), As → As_2_O_3_(g), Cu_2_Se → SeO_2_(g) and Cu_2_Te → TeO_2_(g) is almost independent of temperature.

Out of the impurities present in copper, only Cu_2_Se shows a higher thermodynamic probability of oxidizing to SeO_2_(g) rather than to SeO_2_(l). This trend is also observed in Cu_2_Te when increasing the temperature above 1500 K.

**Figure 4:**
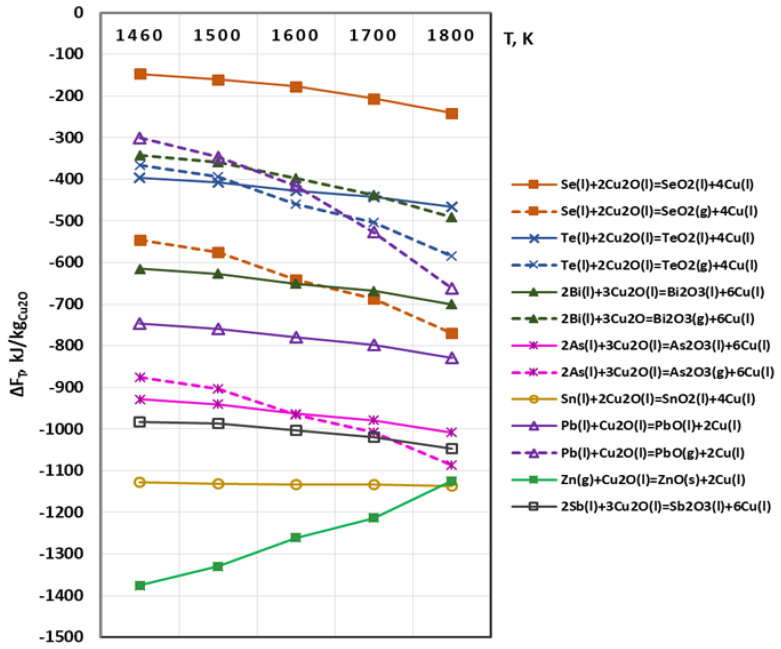
Free energy for interaction between Cu_2_O and metals impurities in vacuum.

The possibility of chemical interactions between Cu_2_O(s,l) and metal impurities is described by Equation (3). The calculated values of ΔF_T_ are shown in Figure 4. The calculations at T = 1460 K were performed under the condition that the copper oxide is in a solid state (Cu_2_O(s)) as the melting temperature of the copper oxide is higher (1508 K).

It is observed that in the studied temperature range all impurities will interact with Cu_2_O regardless of its phase state. The calculated values of ΔF_T_ are high, which indicates a high thermodynamic probability of the process. The chemical interaction of copper oxide with gaseous zinc is most likely to occur in this case as well. Zinc is oxidized to a stable ZnO compound which floats to the reaction surface and Zn will be separated by degassing [20].

The oxidation reactions of Ni and Ag impurities are not shown in Figure 3 and Figure 4, as the calculated values of ΔF_T_ are positive within the investigated temperature range. According to the thermodynamic laws, the course of a given reaction is possible only when the calculated value of the free energy is negative (ΔF_T_ < 0) [27].

The analysis of the reactions with formation of gaseous phase shows that at temperatures above 1600 K, Se, Te and As will be removed mainly in gaseous state, while Sn, Sb, Pb, Bi will be oxidized mainly to oxides–SnO_2_(l), Sb_2_O_3_(l), PbO(l), Bi_2_O_3_(l).

Following the performed thermodynamic analysis, it can be concluded that in the studied temperature range impurities such as Se, Te and As will be oxidized to gaseous oxides, while Sn, Sb, Pb, Bi will be oxidized mainly to liquid oxides.

### 3.2. Refining Efficiency and Microstructures of Obtained Copper

The influence of the temperature (beam power) and the duration of the retention time (τ), during which the melting metal is in liquid state, on the degree of removal of the impurities present in copper technogenic material is evaluated as well. Data about chemical analysis of the impurities concentration of the starting copper material (before EBMR) and of the specimens after e-beam refining of Cu, material losses (estimated using the weight of the initial material and the obtained ingots) and structure of the melted samples are obtained and analyzed under each of the technological regimes studied.

Data for the material losses (W_loss_) which are mainly due to evaporation and also to splashes is presented in Table 1. The results show that the increase of the beam power (temperature) and also the increase of the residence time (τ) lead to an increase of the weight losses W_loss_ (Table 1). The minimum weight loss is 1.63% at T = 1700 K for τ = 20 min.

Data about the changes in the chemical composition of the samples after melting under different EBM technological parameters (regimes) is also presented in Table 1.

**Figure 5:**
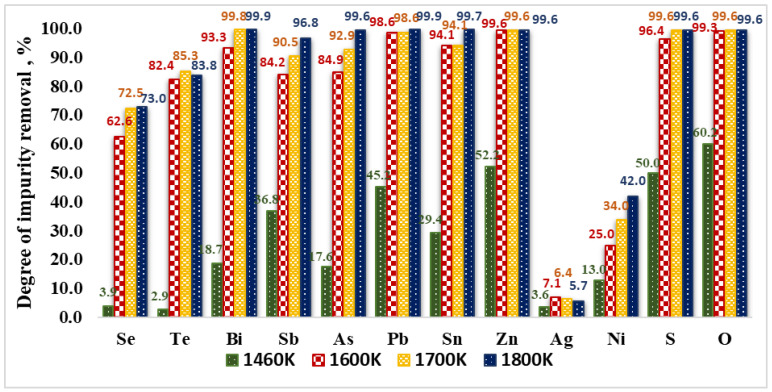
Influence of the temperature on the removal of impurities from copper technogenic material at EBMR.

The influence of the temperature on the degree of impurity removal (α) is presented in Figure 5. The values of the degree of impurity removal are calculated from:

(4) $$\alpha \left(i\right)=\frac{C_{\mathrm{Ri}\left(\mathrm{initial}\right)}-C_{\mathrm{Ri}\left(\mathrm{final}\right)}\;}{C_{\mathrm{Ri}\left(\mathrm{initial}\right)}}\cdot 100\%$$

where C_Ri(initial)_ and C_Ri(final)_ are the initial and final impurity concentrations, respectively.

It can be observed that increasing the temperature from 1460 K to 1600 K leads to the intensive removal of impurities such as Bi, Pb, Sn, Zn and As. The degree of removal of these impurities with the exception of As is more than 93%. In this case, the degree of oxygen removal is 99.3%. This means that during EBM the removal of impurities takes place mainly as a result of the chemical interaction with Cu_2_O. The lower removal values of As (84.9%) and Sb (84.2%) can be explained by the formation of complex compounds between these impurities and Ni (such as strong chalcophyllite 3Cu_2_O·4NiO·Sb_2_O_5_ and antimony arsenate Sb_2_O_3_·As_2_O_5_) during EBMR of copper.

The removal rates (degree of refining) of Se and Te at 1600 K are 62.6% and 82.4%, respectively which can be explained by the fact that these impurities form intermetallic compounds with copper, which are more difficult to oxidize. This fact is consistent with the significantly lower values of the Gibbs energy of the oxidation reactions of these impurities (Figure 3 and Figure 4).

The removal values of oxygen and sulphur at 1600 K are 99.33% and 96.43%, respectively and they increase to 99.56% and 99.64% at 1800 K. Silver and almost all of the nickel remain in copper. Nickel losses can be explained by the total copper losses.

**Figure 6:**
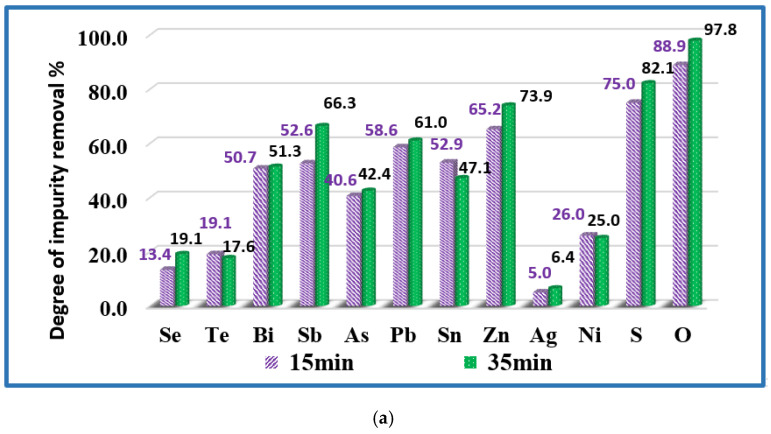

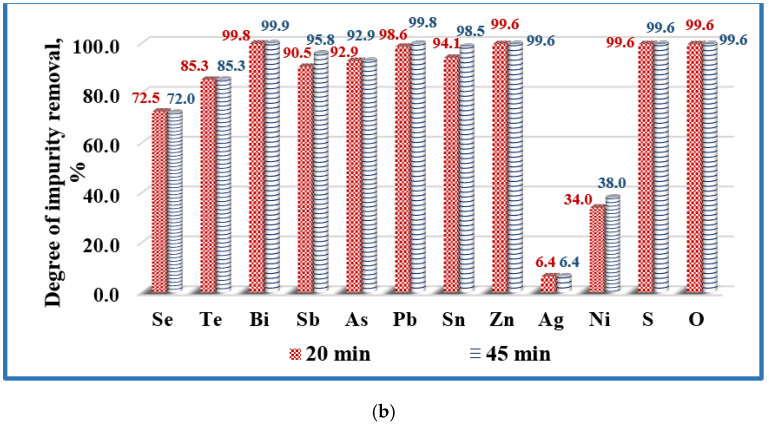
Influence of the melting time on the removal of impurities from copper technogenic material at temperatures: (**a**) T = 1500 K (samples Cu-02 and Cu-03); and (**b**) T = 1700 K (samples Cu-05 and Cu-06).

The influence of the refining time on the degree of impurity removal (α) is evaluated for 1500 K and 1700 K and the calculated values are presented in Figure 6. The analysis of the results shows that at both temperatures, extending the refining time to more than 15–20 min does not significantly affect the degree of removal of impurities from the technogenic copper material. It can be observed that at a temperature of 1700 K and a duration of 20 min the degree of removal of Bi, Pb, Zn is over 98% and that of As, Sb, Sn-about 91–94%. The removal rates of Se (72.5%) and Te (85.3%) are lower. A significant part of Ni and Ag remains in copper.

The highest removal efficiency of oxygen is 99.6% (the minimal oxygen content of 10 ppm) and is obtained at T = 1700 K and T = 1800 K (Figure 5 and Figure 6, Table 1). The optimum of the oxygen refining is connected to higher superheating of the molten metal and better reduction of the oxygen content independently from the retention time in the molten state of the refining copper.

At a temperature of 1500 K extending the refining time increases the rate of sulphur removal from 75% to 82.1%. At higher temperatures, the removal rate is 99.6% regardless of the retention time. The same trend is observed with oxygen.

**Figure 7:**
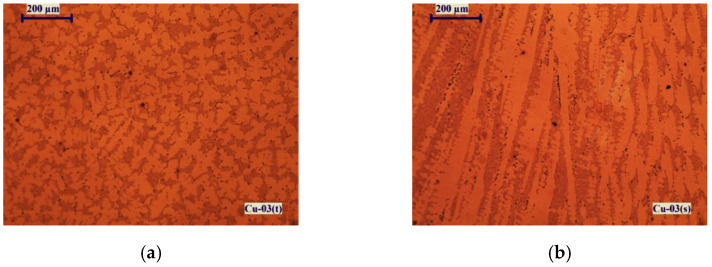
Macrostructures of sample Cu-02 obtained at T = 1500 K for *τ* = 15 min: (**a**) from the top of the ingot; (**b**) from the transverse section.

Figure 7 shows microstructures of the Cu-02 sample manufactured at a temperature of 1500 K for 15 min retention time. The presented structures are from the upper surface of the ingot (top of the ingot)-Cu-02(t) and from the surface along the depth of the ingot (transverse section)-Cu-02(s).

The microstructure observed on the upper surface of the sample (Figure 7a) is dendritic and shows the formation of eutectic melts of type E(Cu-Cu_2_O), E(Cu-Cu_2_S), the presence of loose eutectic melts of insoluble in copper impurities (Pb, Bi) and crystallization of intermetallic phases of Se and Te. The dark stripes in the micrograph of the transverse section of the sample (Figure 7b) show the direction of their crystallization in the volume of copper.

**Figure 8:**
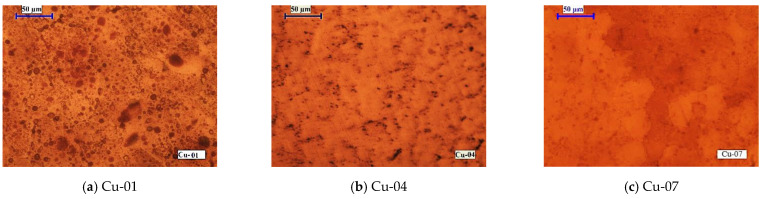

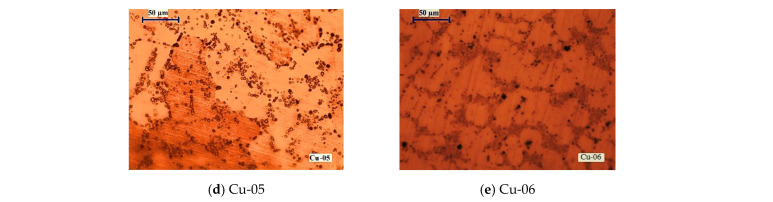
Macrostructures of copper samples manufactured at different EBMR conditions: (**a**) P_b_ = 6 kW, τ = 20 min; (**b**) P_b_ = 10 kW, τ = 20 min; (**c**) P_b_ = 19.5 kW, τ = 20 min; (**d**) P_b_ = 13 kW, τ = 20 min; and (**e**) P_b_ = 13 kW, τ = 45 min.

The effects of the e-beam power and melting time on the microstructures of the copper ingots are presented in Figure 8.

By increasing the beam power (temperature) for 20 min refining time (Figure 8a–d), formation of well-formed globulitic crystals, which are characteristic of nickel-containing copper, is observed. The presence of copper-soluble impurity Ag does not affect the structure. The micrographs in Figure 8d,e show that the extending of the refining time at a given temperature does not affect the microstructure. In this case, the impurity removal rates have similar values.

## 4. Conclusions

The paper investigates the possibility of removing impurities from technogenic copper material (99.45%) using EBM. On the basis of the thermodynamic analysis of the possible chemical interactions occurring in the Cu-Cu_2_O-R_x_ system in the studied temperature range 1460–1800 K and the conducted experimental studies, the influence of the kinetic parameters-temperature (beam power) and melting time on the degree of removal of non-metallic (O, S) and metallic (Se, Te, Pb, Bi, Sn, As, Sb, Zn, Ni, Ag) impurities, the refining efficiency and the structure of the resulting copper was evaluated. The conclusions can be summarized as follows:

- The results obtained show that the electron beam melting method can be successfully applied for refining copper technogenic material with a high content of impurities, which in the conditions of EBM are in a gaseous state (such as Bi, Pb, Zn, As, Sb, Sn) and reach nearly 100% removal degree and ~97% for Sb.
- Oxygen and sulphur also reach a high degree of removal (≥99%). Under the studied conditions, the maximum degree of refining of Se and Te is 73% and 85.3%, respectively, which is due to the fact that Se and Te form intermetallic compounds with copper, which are more difficult to oxidize. This corresponds to significantly lower values of the Gibbs energy of the oxidation reactions of these impurities.
- Silver and most of the nickel remain in copper. Under vacuum conditions and at the temperature range studied, silver does not oxidize or evaporate. The low degree of refining of nickel (34–42%) from copper can be explained by the good solubility of this impurity in copper. In addition, nickel is also not oxidized in the studied temperature range. It was found that raising the temperature above 1700 K, as well as extending the melting time over 20 min hardly change the purity and structure of the resulting refined metal.
- At temperatures in the range 1600–1800 K, the achieved refining efficiency is 78–85% and the purity of copper after EBM is 99.9%. The highest total refining efficiency of 84.6% is seen at a beam power of 19.5 kW for 20 min melting time and the best purification of copper technogenic material (99.92%) is achieved.

## Figures and Tables

**Table 1:** Concentration of impurities at electron beam processing of copper technogenic material.

Probe	Cu-00	Cu-01	Cu-02	Cu-03	Cu-04	Cu-05	Cu-06	Cu-07
Concentration before EBMR				Process Parameters				
Type	ppm	T = 1460 K	T = 1500 K		T = 1600 K	T = 1700 K		T = 1800 K
		t = 20 min	t = 15 min	t = 35 min	t = 20 min	t = 20 min	t = 45 min	t = 25 min
Se, ppm	382	367	331	309	143	105	107	103
Te, ppm	68	66	55	56	12	10	10	11
Bi, ppm	150	122	74	73	<10	<1	<1	<1
Pb, ppm	210	115	87	82	<3	<3	<1	<1
Sn, ppm	34	24	16	18	<2	<2	<1	<1
Sb, ppm	95	60	45	32	15	<9	<4	<3
As, ppm	700	577	416	403	106	50	50	<3
Ni, ppm	1000	870	740	750	750	660	620	580
Zn, ppm	23	11	8	6	<1	<1	<1	<1
Ag, ppm	140	135	133	131	130	131	131	132
O, ppm	2251	897	250	50	15	10	10	10
S, ppm	28	14	7	5	<1	<1	<1	<1
Cu,%	99.454	99.675	99.784	99.809	99.892	99.902	99.907	99.916
^1^*ε_tot_*,%		40.5	60.4	65.0	78.3	82.1	83.0	84.6
W_loss_,%		0.42	0.86	2.07	1.63	2.93	3.56	3.06

^1^*ε_tot_*-total efficiency of copper refining.
